# Emerging Role of Splenic Macrophage in Malaria Pathogenesis and Immunity

**DOI:** 10.1002/iid3.70258

**Published:** 2025-10-31

**Authors:** Aarti Gupta, Meenu Kalkal, Jyoti Das

**Affiliations:** ^1^ Division of Immunology ICMR‐National Institute of Malaria Research Dwarka New Delhi India; ^2^ Academy of Scientific and Innovative Research (AcSIR) Ghaziabad Uttar Pradesh India

**Keywords:** CD47, cytokines, macrophage, malaria, phagocytosis, polarization, SIRPα

## Abstract

**Background:**

Malaria, caused by *Plasmodium* parasites, remains a leading global health concern, impacting millions of people globally. Splenic macrophages are specialized immune cells that reside in the spleen and play a pivotal role in the pathogenesis and immune response during malaria. These cells are fundamental to the body's defense against *Plasmodium* due to their multifaceted roles in parasite clearance, antigen presentation, and immune regulation. They participate in the early immune response by phagocytosing infected red blood cells, producing cytokines, and interacting with other immune cells to modulate both pro‐inflammatory and anti‐inflammatory responses.

**Aim:**

This review highlights the evolving understanding of splenic macrophages in malaria pathogenesis and immunity and underscores their importance in the development of novel therapeutic interventions.

**Method:**

The search strategy included retrieving relevant studies from PubMed Central and Google Scholar spanning between 1955–2025. Articles were screened based on relevance to malaria immunology, macrophage biology, and therapeutic interventions. Both experimental and clinical studies were considered, and references from selected articles were also cross‐checked to identify additional relevant literature. Priority was given to peer‐reviewed articles, systematic reviews, and original research that provided insights into the immunological role of splenic macrophages in malaria.

**Result:**

Splenic macrophages play a dual role in disease progression and severity. They have a specialized ability to polarize into different functional states under different microenvironments. Notably, macrophage polarization during *Plasmodium* infection is not strictly dichotomous but exists along a continuum, with macrophages exhibiting both M1 and M2 characteristics, facilitating dynamic immune modulation. This continuum of polarization is essential for balancing immune responses and ensuring effective immunity while avoiding excessive inflammation. Emerging evidence suggests that splenic macrophages significantly influence malaria severity and clinical outcomes, positioning them as key targets for therapeutic strategies.

**Conclusion:**

Optimizing macrophage‐mediated immunity by targeting specific macrophage functions and biomarkers could hold promise for improving vaccine development, diagnostics, prognosis, and treatment strategies, thereby enhancing clinical outcomes for malaria patients.

## Summary

- Splenic macrophages play a crucial role in providing immunity during malaria pathogenesis.
- Different *Plasmodium* strains have different impact on splenic macrophages during infection.
- Splenic macrophages contribute toward immune defense through various mechanisms such as by engaging in phagocytosis, facilitating ADCI (antibody‐dependent cellular inhibition) and through release of cytokines.
- Emphasizing the role of splenic macrophages in cellular and humoral immune response could enhance strategies for effective vaccine development.
- Enhancing macrophage‐mediated immunity can lead to advancements in diagnosis, prognosis, and treatment approaches for malaria.

**Abbreviations**
ADCI: antibody‐dependent cellular inhibition
BM: bone marrow
CD47: cluster of differentiation 47
DAMPs: damage‐associated molecular patterns
GM‐CSF: granulocyte–macrophage colony‐stimulating factor
GPI: glycosylphosphatidylinositol
HO‐1: heme‐oxygenase 1
HSPs: heat‐shock proteins
IL‐3: interleukin‐3
KCs: Kupffer cells
MCP: monocyte chemoattractant protein
MIF: macrophage migration inhibitory factor
MMZM: metallophilic marginal zone macrophages
MZM: marginal zone macrophages
NLR: NOD‐like receptor
PAMPs: pathogen‐associated molecular patterns
pDC: plasmacytoid dendritic cells
PPAR‐ γ: peroxisome proliferator‐activated receptor‐γ
PRRs: pattern‐recognition receptors
RBCs: red blood cells
RPMs: red pulp macrophages
SIRPα: signal inhibitory regulatory protein alpha
T1‐IFNs: Type I interferons
TRMs: tissue resident macrophages

## 1 Introduction

Globally, malaria remains a significant cause of illness and mortality, especially in tropical areas, and presents a substantial public health challenge. In malaria, an effective control of infection by the immune system is ensured by the well‐organized architecture of the secondary lymphoid organ, specifically the spleen [1]. The spleen is a well‐organized organ composed of connective and lymphoid tissues, featuring an intricate compartmentalization with a complex microcirculatory system. Spleen primarily serves as a pivotal site for both the filtration of blood and the orchestration of immune responses [2]. The microarchitectural design of the spleen's filtering function is intricately linked to its trabecular framework, which comprises three primary components: (a) the white pulp, a lymphoid tissue that houses a significant proportion of immune effector cells; (b) the red pulp, characterized by a reticular meshwork responsible for the removal of aged and abnormal red blood cells (RBCs), as well as *Plasmodium* infected RBCs (iRBCs); and (c) the marginal zone, situated between the white and red pulp, where the clearance of inert particles, bacteria, and viruses occurs [2]. During *Plasmodium* infection, the spleen plays a critical role in the body's defense against malaria, serving as a primary site for filtering iRBCs and coordinating immune responses by regulating immune cell interactions, antigen presentation, and the modulation of inflammatory responses [3]. Within this context, splenic macrophages are key players in initiating and regulating immune responses to *Plasmodium* infection [4, 5]. These specialized myeloid cells perform several essential functions, including phagocytosis of iRBCs, cytokine production, and antigen presentation to T cells, all are crucial for controlling infection and maintaining immune homeostasis [4]. During *Plasmodium* infection, various splenic macrophage subsets, such as red pulp macrophages (RPMs), marginal zone macrophages (MZM), and metallophilic macrophages, play an important role in anti‐plasmodial activity [6]. These subsets of splenic macrophages play a vital role in defending against malarial parasites through various mechanisms and distinct targets. For immunity, they phagocytose *Plasmodium* iRBCs via opsonization and pattern recognition, present parasite antigens to T cells for adaptive immune activation, detoxify hemozoin to prevent immune suppression, and secrete pro‐inflammatory cytokines to enhance parasite clearance [7]. To mitigate severity, splenic macrophages also regulate inflammatory response by balancing pro‐ and anti‐inflammatory cytokines (avoid cytokine storms or immune suppression). Additionally, they also recycle iron from erythrocytes to support erythropoiesis, preventing anemia and oxidative stress, while promoting tissue repair and helps in maintaining splenic architecture to ensure sustained immune function during chronic infections [7]. Therefore, for the better understanding of the functional heterogeneity and plasticity of these macrophage subsets during *Plasmodium* infection is much needed area of research.

Recent developments have reinforced the concept that an effective host immune response is crucial for developing a potent vaccine that can significantly contribute to the control, prevention, and elimination of malaria. Despite global efforts made to develop a highly efficacious malaria vaccine, the search remains challenging because of the multi‐stage life cycle and genetic diversity of malaria parasite [8]. After worldwide tremendous effort made for the development of effective malaria vaccines, World Health Organization (WHO) has given approval for clinical use of adjuvant vaccines like RTS, S/ASO1 (Mosquirix) [9], and R21/Matrix‐M (R21/MM) [10]. These vaccines are the pre‐erythrocytic stage malarial vaccines which specifically target the *Plasmodium falciparum* circumsporozoite protein or antigen and aim to provide safe and effective protection against malaria. Still, the clinical efficacy of these vaccines is very limited and can provide protective immunity for a short duration in children [11, 12, 13]. However, advancements in comprehending the host immune response to different strains and stages of the *Plasmodium* parasite have led to the identification and characterization of certain crucial vaccine candidates [14]. Thus, cell base immunotherapies can be effective strategies with a focus on both inducing humoral and cellular immune responses for an effective protective immunity. Therefore, splenic macrophages can be targets as chemotherapeutics, through the processes, such as by enhancing iRBC clearance, modulating macrophage polarization, or improving antigen presentation, which holds promise for improving malaria outcomes by both enhancing immunity and mitigating pathological damage. Therefore, extensive research needs to be made toward creating a deployable, effective malaria vaccine in the medium‐to long‐term, with focusing on both inducing humoral and cellular immune responses for an effective protective immunity.

The search strategy used for this review article includes PubMed Central and Google Scholar searches between 1955 and 2025 as shown in Supporting Information S1: Figure, with search terms developmental origins and differentiation of splenic macrophages, splenic macrophage subtypes in malaria, splenic macrophage phenotypes in malaria, *Plasmodium* strain impact on splenic macrophages, splenic, macrophage in erythropoiesis and malarial anemia, innate immune activation by splenic macrophages during malaria, splenic microarchitecture and myeloid cell dynamics in malaria, circadian regulation of macrophage responses in malaria, M1/M2 macrophage polarization in malaria, natural compounds modulating macrophage polarization during malaria, erythrophagocytosis by splenic macrophages during malaria, CD47‐SIRP‐alpha in malaria, macrophage‐based immunotherapies in disease including malaria.

## 2 Understanding Developmental Origins and Differentiation of Splenic Macrophages

Macrophages are myeloid cells, and are the integral part of the mononuclear phagocyte system (MPS). They are first line of defense against any foreign infection, alongside other innate cells of immune system. They can be broadly categorized into bone marrow (BM)‐derived macrophages (BMDMs) and tissue‐resident macrophages (TRMs), each with distinct roles depending on their origin and location [15, 16] as shown in Table 1. BMDMs are generated from hematopoietic stem cells in the BM, with monocytes released into the bloodstream and differentiating into macrophages at sites of infection or injury. These short‐lived macrophages are highly plastic, adopting pro‐inflammatory (M1) or anti‐inflammatory (M2) phenotypes based on environmental cues, and are crucial for acute immune responses, pathogen clearance, and tissue repair. Conversely, TRMs are long‐lived macrophages that arise either from embryonic progenitors, such as yolk sac or fetal liver‐derived cells, or from monocytes in adulthood. They are strategically located in specific tissues, where they adapt to local microenvironments and perform specialized functions, such as RBC recycling by RPMs in the spleen, detoxification by Kupffer cells (KCs) in the liver, and neuronal support by microglia in the brain [17, 18]. Splenic macrophages, classified as TRMs, exhibit a variety of origins and differentiation pathways influenced by their environment, thereby influencing their transcriptional profiles and activation states [19, 20]. In mice, splenic macrophages have a dual origin; approximately 55% of the spleen macrophage population is supplied by monocyte influx, while 45% is derived from local production within the spleen [21]. During TRM development, the role of the microenvironment and involvement of several transcription factors are crucial for their reprogramming ability [22]. Additionally, the transcription factors Maf and Maf‐b play important roles in regulating macrophage differentiation and proliferation [22]. To fully delineate the transcriptional events governing macrophage biology, numerous studies have investigated the genome‐wide distribution of enhancers and key regulatory factors. PU.1 is recognized as a principal regulator of macrophages, occupying a significant number of enhancers [23]. Beyond these master regulators, distinct macrophage populations rely on specific transcription factors for their development and differentiation. For instance, the transcription factor Gata‐6 is critical for the differentiation of peritoneal macrophages [24] while the nuclear receptor PPAR‐γ is vital for maintaining alveolar macrophage identity [25]. In the spleen, the transcription factors Spic and IRF‐8 are instrumental in the development of RPMs [26, 27, 28], whereas LXR‐α is essential for differentiating the marginal zone and metallophilic macrophages [29]. These transcriptional regulators highlight the tissue‐specific nature of macrophage differentiation, demonstrating that the unique microenvironment of the spleen drives the development of distinct macrophage subtypes populations that play critical roles in immune surveillance, pathogen clearance, and tissue homeostasis. Understanding these regulatory pathways offers valuable insights into macrophage biology and may inform therapeutic strategies targeting spleen‐resident macrophages in various diseases.

**Table 1:** Summarizes the distinct characteristics and roles of splenic macrophages and bone marrow‐derived macrophages.

Feature	Splenic macrophages	Bone marrow‐derived macrophages (BMDMs)
Origin	Primarily from embryonic progenitors (yolk sac or fetal liver); partially from monocytes	Derived from hematopoietic stem cells (HSCs) in the bone marrow via monocyte influx
Localization	Reside exclusively in the spleen	Recruited to sites of infection, injury, or inflammation
Subtypes	Red pulp macrophages, marginal zone macrophages, and metallophilic macrophages	Not specialized; differentiate based on environmental cues
Function	– Maintain blood homeostasis and recycle red blood cells – Respond to blood‐borne pathogens – Support adaptive immunity	– Acute immune response – Inflammation and tissue repair – Pathogen clearance
Plasticity	Adapted to splenic microenvironment, limited phenotypic plasticity	High plasticity; can polarize into M1 (pro‐inflammatory) or M2 (anti‐inflammatory) phenotypes
Lifespan	Long‐lived and self‐renewing within the spleen	Short‐lived; continuously replenished by monocytes from the bone marrow
Transcriptional regulation	– Spi‐c and IRF‐8 regulate red pulp macrophages – LXR‐α governs marginal zone and metallophilic macrophages	– PU.1, Maf, and Mafb regulate differentiation and activation

### 2.1 Functional Specialization of Splenic Macrophage Subtypes in Malaria Pathogenesis

In the spleen, macrophage heterogeneity has been long documented, with four subsets populating the different splenic compartments with specialized functions and locations within the spleen. They are as RPMs situated in the red pulp, MZM and marginal zone metallophilic macrophages found within the marginal zone, and tangible body macrophages in white pulp [6]. The splenic macrophage subtypes in both murine and human models exhibit significant diversity, reflecting their specialized roles in innate immune functions, red blood recycling, iron homeostasis, and response to amyloid deposition, and so forth [2, 3, 6, 30] as shown in Table 2. This diversity and functionality are basically derived from arising anatomical and functional differences between the mouse and human spleen due to species‐specific traits, which significantly impact the interpretation of immunological studies. Human and mice spleen are primarily different in three aspects‐ (i) In the human spleen, the marginal zone does not exhibit a distinctly defined marginal sinus and is instead encircled by an additional perifollicular zone. This zone contains blood vessels that terminate in capillaries, facilitating a rapid “closed” microcirculation that bypasses the filtration cords. (ii) The human spleen is characterized as sinusoidal, in contrast to the non‐sinusoidal nature of the mouse spleen. (iii) Notably, while the mouse spleen exhibits significant erythropoiesis, such activity has not been documented in the human spleen [5, 31, 32]. Furthermore, the human spleen has limited capacity for active contraction, unlike the mouse spleen, which can expel RBCs during stress [33].

**Table 2:** Murine and human splenic subtypes associated with different functions and limitations during *Plasmodium* infection.

Sl. no	Subtypes	Murine	Human
1.	Red pulp macrophages	Specialized in clearing senescent and infected erythrocytes. Recycle iron efficiently [5, 30].	Similar functions, but lower activity in malaria. Also process heme and detoxify iron [1, 30, 34].
2.	Marginal zone macrophages	Highly abundant; robust in recognizing blood‐borne pathogens via scavenger and complement receptors [5, 30, 35].	Present but less distinct. Functions overlap with other macrophage subsets [6, 31].
3.	White pulp macrophages	Support adaptive immunity by interacting with T and B cells [1, 21, 30, 36].	Play a role in T‐cell activation but are less studied compared to murine counterparts [30, 31].

Considering these differences in human spleen, most of the human studies often rely on ex vivo or in vitro analyses of blood monocytes due to difficulty in accessing human macrophages in these tissues, which may not fully capture the complexities of splenic immune responses observed in mice. Thus, for the better understanding of splenic macrophages subtypes, murine models provide a versatile and advancing valuable insights that help to understand the necessity of extrapolating findings to human health and disease.

**Figure 1:**
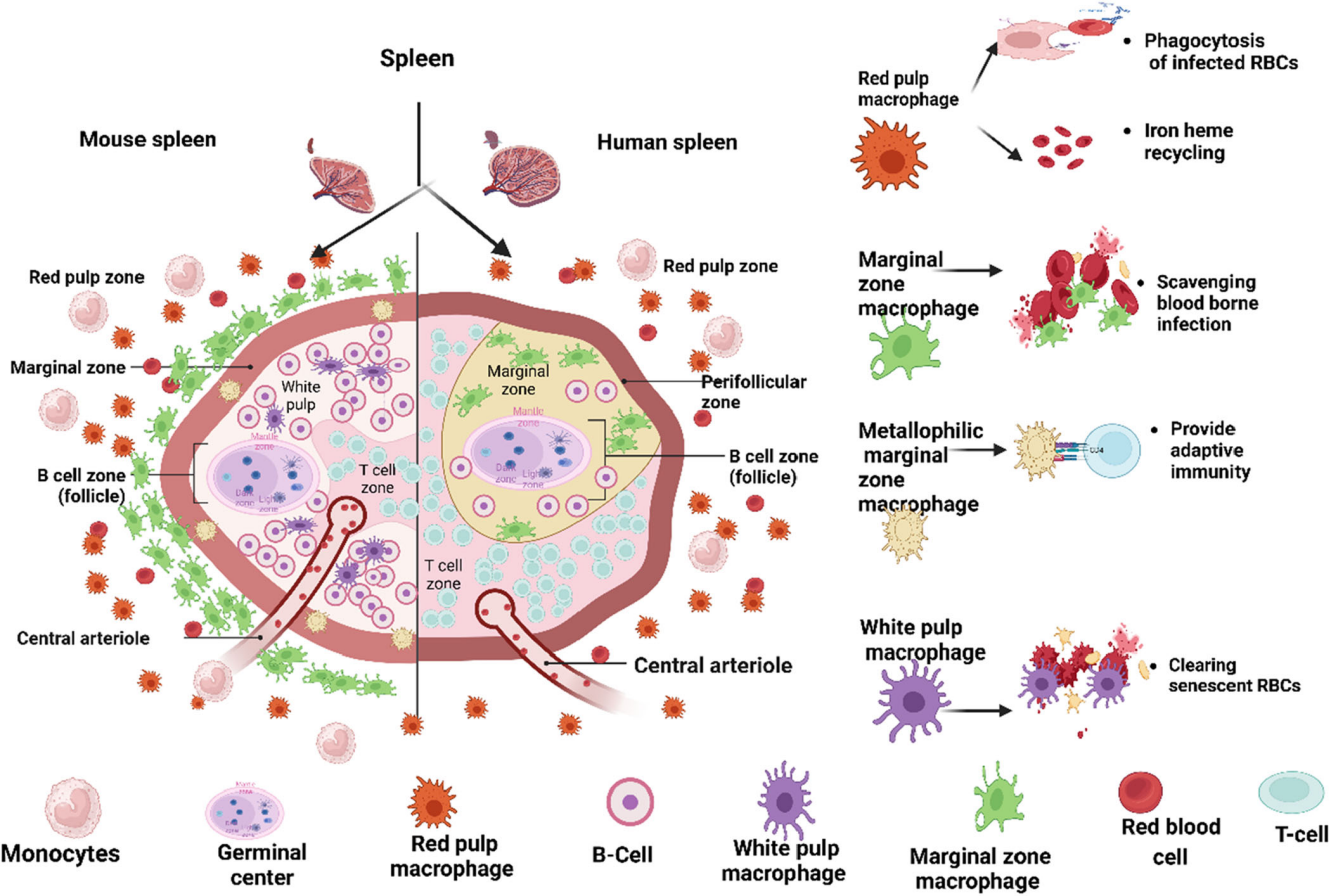
Comparative localization and functional specialization of splenic macrophage subsets in mouse and human spleens during malaria pathogenesis. (Created with Bio Render.com.)

Splenic macrophage subtypes in murine, having metallophilic marginal zone macrophages (MMZM) and MZM possess an ability to engulf blood‐borne pathogens like viruses and bacteria. Their strategic positioning near splenic regions rich in T and B cells facilitates quick interactions between these macrophages and cells involved in adaptive immunity [30]. Indeed, MMZM and MZM serve as crucial connectors between the innate and adaptive immune systems, while RPMs are traditionally seen as responsible for clearing aging RBCs [37]. Research on different strains of mice infected with *Plasmodium berghei* K173 demonstrated that splenic RPMs are key in controlling an individual's total parasite burden, affecting the progression of malaria [37]. In contrast, human splenic macrophage subtypes exhibit fewer anatomically distinct subpopulations compared to mice, characterized by overlapping functions and greater reliance on circulating monocytes. The white pulp macrophages are a major operator of the humoral immune response, especially to circulating antigens. Meanwhile, the RPMs exert a unique and subtle control over the surface integrity and biomechanical properties of RBCs [38]. Understanding these differences is crucial for highlighting the advantages and limitations of murine models in fully replicating human malaria pathology emphasizes the need for further characterization of human splenic macrophage subtypes and their functions for developing targeted therapies in immune‐related diseases (Figure 1).

This schematic representation of splenic architecture in mouse (left) and human (right) highlights the distribution and immunological roles of distinct macrophage subsets within the red pulp, white pulp, and marginal zone. RPMs, localized in the red pulp zone, are primarily involved in phagocytosis of *Plasmodium* iRBCs and iron‐heme recycling. MZM, situated in the marginal zone, play a critical role in capturing and clearing blood‐borne pathogens. MMZM, located near the marginal sinus, are implicated in antigen presentation and bridging innate to adaptive immune responses. White pulp macrophages, confined to the white pulp, contribute to the clearance of senescent erythrocytes and primarily support innate immunity.

### 2.2 Phenotypes of Splenic Macrophages in Malaria Pathogenesis

Immune cells exhibit diverse phenotypes, characterized by specific sets of marker molecules and expression patterns, where plus sign (+) denotes positive expression, while a minus sign (−) indicates low or no expression. This immunophenotype primarily reflects protein‐level expression [39]. Each splenic macrophage is known to exhibit a unique phenotype. RPMs express F4/80, α9 integrin, and vascular cell adhesion molecule‐1(VCAM‐1) [40]. RPMs, often referred to as “reservoir monocytes,” are characterized as Cx3cr1 promoter‐driven GFP^+^ F4/80^+^/^low^ macrophages. These cells possess an ability to exit the spleen and migrate to inflamed tissues in response to inflammatory processes, regardless of their location [41]. In the marginal zone, MZM are present at the outer marginal zone, while marginal zone metallophilic macrophages are present at the inner marginal zone, express SIGN‐R1 and CD169, respectively [18]. Furthermore, tangible body macrophages found in the germinal centers of B‐cell follicles in the white pulp express CD68 and milk fat globule‐epidermal growth factor‐8 [18, 42]. In humans, monocytes can be categorized into three primary subsets according to the expression of the surface markers CD14 and CD16 [43]. In the bloodstream of a healthy adult, around 90% of the total monocyte population consists of classical monocytes, which are characterized or described as CD14^bright^CD16^−^. The remaining 10% comprises as CD16^+^ monocytes, which can be subdivided into intermediate or inflammatory monocytes (CD14^bright^CD16^+^) or nonclassical monocytes (CD14^dim^CD16^+^). These subsets have the potential to increase in number during instances of infection and inflammation [43]. Various studies have reported the expansion of these monocyte subsets in patients with malaria [44, 45, 46, 47, 48]. Therefore, understanding the significance of phenotype surface markers opens up new avenues for exploring the complexities of cellular biology and its implications across diverse scientific disciplines.

*Plasmodium* infection has a significant impact on the phenotype of splenic macrophages. Various phenotypic markers like CD169, CD14, F4/80, T‐cell immunoglobulin and mucin‐domain‐containing molecule 3 (Tim‐3), CD11b, and so forth are commonly observed in mice splenic macrophages. Studies have shown that *Plasmodium* infection leads to changes in the expression of various surface molecules or markers present on macrophages, influencing their function during malaria pathogenesis [49]. CD169, an immune macrophage marker, is a member of the Siglec family [50]. CD169^+^ macrophages have been predominantly identified in various regions of secondary lymphoid organs, including the metallophilic marginal zone (MZM) of the spleen, the subcapsular sinus, and the medulla of lymph nodes. These macrophages can directly interact with T cells, B cells, and dendritic cells (DC) via the CD169 molecule, thereby playing a significant role in immune regulation [51, 52]. In a study conducted by Gupta et al., in 2016, where they used the transgenic BALB/c mouse model by depleting CD169 through the administration of diphtheria toxin, then subsequently infected the mice with *P. berghei* ANKA. They observed that CD169^+^ played a critical role in restricting parasite sequestration to organs such as spleen, liver, kidney, and lungs. Without this regulation, there is a risk of systemic inflammation and damage to multiple organs [49]. Thus, CD169^+^ macrophages are crucial to mitigating *Plasmodium* infection and pathogenesis by limiting the infection‐induced inflammation.

In addition, another important surface marker F4/80, expressed on mouse macrophages, plays a crucial role in the formation of antigen‐specific regulatory T cells (Tregs), which are vital for maintaining immune tolerance and immunosuppression [53, 54]. Therefore, an elevation in F4/80 expression may enhance malaria‐induced immune suppression by facilitating Treg activity [55, 56]. RPMs have been associated with the regulation of blood‐stage malaria [3]. In addition, a phenotypic characteristic shared by RPMs and DCs was observed in a portion of splenic red pulp with strong F4/80+ and CD11c labeling during *Plasmodium chabaudi* infection [57]. This population plays a crucial role in the early clearance of *P. chabaudi* parasites, but it sharply declines during high peak of parasitemia. RPMs have a slow turnover rate and may undergo cell death after ingesting *Plasmodium* iRBCs due to the toxic effects of hemozoin. These findings suggest that targeting F4/80+ macrophages or their interaction with DCs could offer a promising approach for modulating immune responses in malaria. Furthermore, CD11b, also known as integrin αM, which is a subunit of the integrin complex Mac‐1 (CD11b/CD18), which is primarily found on myeloid cells, including macrophages, neutrophils, and DCs. CD11b plays a critical role in innate immune responses, inflammation, and the resolution of infection [58]. Moreover, CD14, a cell surface antigen primarily associated with macrophages and monocytes, is not only crucial for immune responses but also has diverse functions in various nonimmune cell types, highlighting its potential impact on broader physiological processes [59]. In another study using C57BL/6 mice infected with *P. berghei* ANKA, a novel population of CD11b^high^CD14^+^F4/80^+^ macrophages expressing a unique combination with TCR‐β was observed during infection. These cells were primarily located in the brain and spleen with an observable expansion of TCR‐β, and exhibited a remarkable ability to engulf parasitized RBCs during experimental cerebral malaria. Hence, the expansion of TCR‐β expressing CD11b^high^CD14^+^F4/80^+^ macrophage converges both innate and adaptive immune response to modulate the host response against malaria and other infection [60]. However, whether TCR activation directly promotes phagocytosis in macrophages remains unclear.

In addition, another macrophage marker, T‐cell immunoglobulin‐ and mucin‐domain‐containing molecule 3 (Tim‐3), has emerged as an important regulatory molecule in cell‐mediated immune response [61] during *Plasmodium* infection. Initially recognized as being expressed on CD4^+^ Th1 and CD8^+^ T‐cytotoxic type 1 cells, Tim‐3 plays a complex role in immune modulation and regulates the distinct function in macrophages [62, 63]. In all murine malarial parasite infections, mice depleted of or lacking CD4^+^ T cells cannot clear the parasites [64, 65, 66]. In conjunction with PD‐1, it support the promotion or sustenance of Th1 cells response in the early stages of blood‐stage *Plasmodium yoelii* [67]. However, Tim‐3 also negatively regulates the immune response, with studies showing that blocking Tim‐3 signaling can enhance sterile immunity during malaria. A study in which monocytes from individuals infected with *P. falciparum* and splenic macrophages of mice infected with *P. berghei* exhibit an increase in the population of monocytes and macrophages, which subsequently exhibit reduced levels of Tim‐3, an immunomodulatory molecule. This reduction ultimately leads to a decreased or suppressed anti‐malarial response [68]. Although Tim‐3 hinders the ability of CD11b^+^ splenic macrophages in mice from engulfing *P. berghei*‐infected cells, the blockade of Tim‐3 in these malaria‐infected mice restored lymphocyte activity. It also improved the phagocytosis by macrophages and an enhanced production of TNF*α*, inducible nitric oxide synthase (iNOS), and IL‐12. Additionally, Tim‐3 is also well recognized for its association with removal of apoptotic cells through interaction with phosphatidylserine. A recent study identified a novel phagocytic receptor for infected cells named as Tim‐4 during malaria infection. Such infected RBCs are phagocytosed in the spleen via the interaction between phosphatidylserine present on infected RBCs and the Tim‐4 receptor on splenic macrophages [69]. In conclusion, Tim‐3 serves as both a promoter and a suppressor of immune responses during malaria infection, suggesting that targeting Tim‐3 and its related pathways could be a therapeutic strategy to enhance immune clearance and control the progression of malaria.

## 3 Impact of Different Plasmodium Strains on Splenic Macrophages in Malaria Pathogenesis

Different *Plasmodium* strains exhibit distinct effects on splenic macrophages, influencing immune responses and disease outcomes. Research studies indicate that the interactions between various *Plasmodium* strains and splenic macrophages are complex and strain‐specific, leading to diverse immunological consequences and mechanisms [70, 71]. Therefore, understanding these interactions is crucial for developing targeted therapeutic strategies and vaccines against malaria. In murine malaria pathogenesis, a study was conducted on experimental infections with nonlethal (17X) and lethal (17XL) *P. yoelii* strains, in which the higher parasite accumulation, reduced motility, loss of directionality, increased residence time, and altered magnetic resonance were observed only in the spleens of mice infected with 17X but not with 17XL. These differences were linked to the formation of a strain‐specific spleen tissue barrier of fibroblastic origin. This barrier was associated with evasion of RPM‐mediated clearance and the adherence of infected RBCs to the barrier, highlighting a unique pathogenic mechanism [70]. In nonlethal malaria, the barrier systems effectively shield splenic reticulocytes from being infected by parasites. Conversely, in the case of lethal 17XL malaria, these protective mechanisms fail, leading to a significant rise in parasitization within the spleen, accompanied by an increase in the activity of macrophages. The extensive parasitization and subsequent recycling of parasites through the abundant splenic reticulocyte reserves observed in lethal malaria, contrasted with the preservation of these reticulocyte reserves in nonlethal malaria, indicates that the spleen's functions exacerbate the severity of lethal malaria while mitigating the effects of nonlethal malaria [71]. These findings imply that the dynamics of reticulocyte involvement in nonlethal rodent malaria differ from those observed in other *Plasmodium* species, thereby paving the way for further functional and structural investigations of the spleen in the context of malaria.

In addition, *P. berghei* K173, exhibits varying impacts on splenic macrophages depending on the genetic background of the host. *P. berghei* K173‐infected C57BL/6 mice showed a significant increase in macrophage percentages, correlating with lower parasitaemia. Conversely, ICR mice exhibited reduced macrophage levels and higher parasitaemia. KM mice also showed an increase in macrophage percentages; however, they experienced structural disorganization within the spleen, which impaired their capacity to regulate parasitaemia. These findings underscore the critical role of genetic background in shaping splenic macrophage responses to malaria infection, influencing disease progression and outcomes [37]. In another study, the expansion of splenic macrophages was studied using two different strains of *Plasmodium*: *P. yoelii* and *P. berghei*. As a result, they observed that infection with *P. yoelii* resulted in an earlier appearance and higher numbers of adherent splenic macrophages compared to infection with *P. berghei* [72]. Additionally, macrophages from *P. yoelii*‐infected mice produced significantly higher levels of O_2_ metabolites (H_2_O_2_ and O_2_^−^) than those from *P. berghei*‐infected mice, indicating that lethal and nonlethal malaria species differentially influence macrophage activity and the overall disease outcome [72]. Additionally, some studies show that during an infection with *P. chabaudi*, there is a significant loss of splenic macrophages population, especially pronounced in the acute phase of the infection [73]. During peak parasitemia, both metallophilic marginal zone macrophages (MMZM) and marginal zone macrophages (MZM) are completely lost. This process is driven by CD8^+^ T cells through specific death pathways that are independent of inflammatory cytokines but rely on key signals such as CD95 and perforin [73]. Therefore, iRBCs are not sequestered or phagocytosed by macrophages located in the marginal zone; instead, they are directly filtered for phagocytosis by RPM in the red pulp zone [74]. While some studies have examined the effects of human host *Plasmodium* strains—*P. falciparum* and *Plasmodium vivax* on splenic microarchitecture explaining disturbances/disorganization in spleen during infection [38, 75, 76]. However, there is gap in literature on how these parasites specifically influence splenic macrophages. Thus, there is a need for further studies to elucidate how different *Plasmodium* strains modulate splenic macrophage populations and functions in malaria.

In parallel to these findings from murine studies, murine models have provided critical insights from time to time into the pathogenesis of malaria in humans by exploring host genetics, immune responses, and mechanisms of virulence. *Plasmodium* infections in mice are influenced significantly by the genetic background of the host, with findings often correlating with human studies due to the high genetic homology between mice and humans [77]. Additionally, humanized mice models are employed to replicate human malaria infections, enabling detailed exploration of the intricate interactions between the host and the *Plasmodium* parasite [78]. Therefore, murine models have been instrumental in bridging preclinical research with human clinical trials. They have enhanced understanding of the pharmacokinetics and pharmacodynamics of antimalarial drugs [79]. This translational approach accelerates the development of effective treatments by providing critical data that align with human responses.

## 4 Functional Roles of Splenic Macrophages in Malaria Pathogenesis

**Figure 2:**
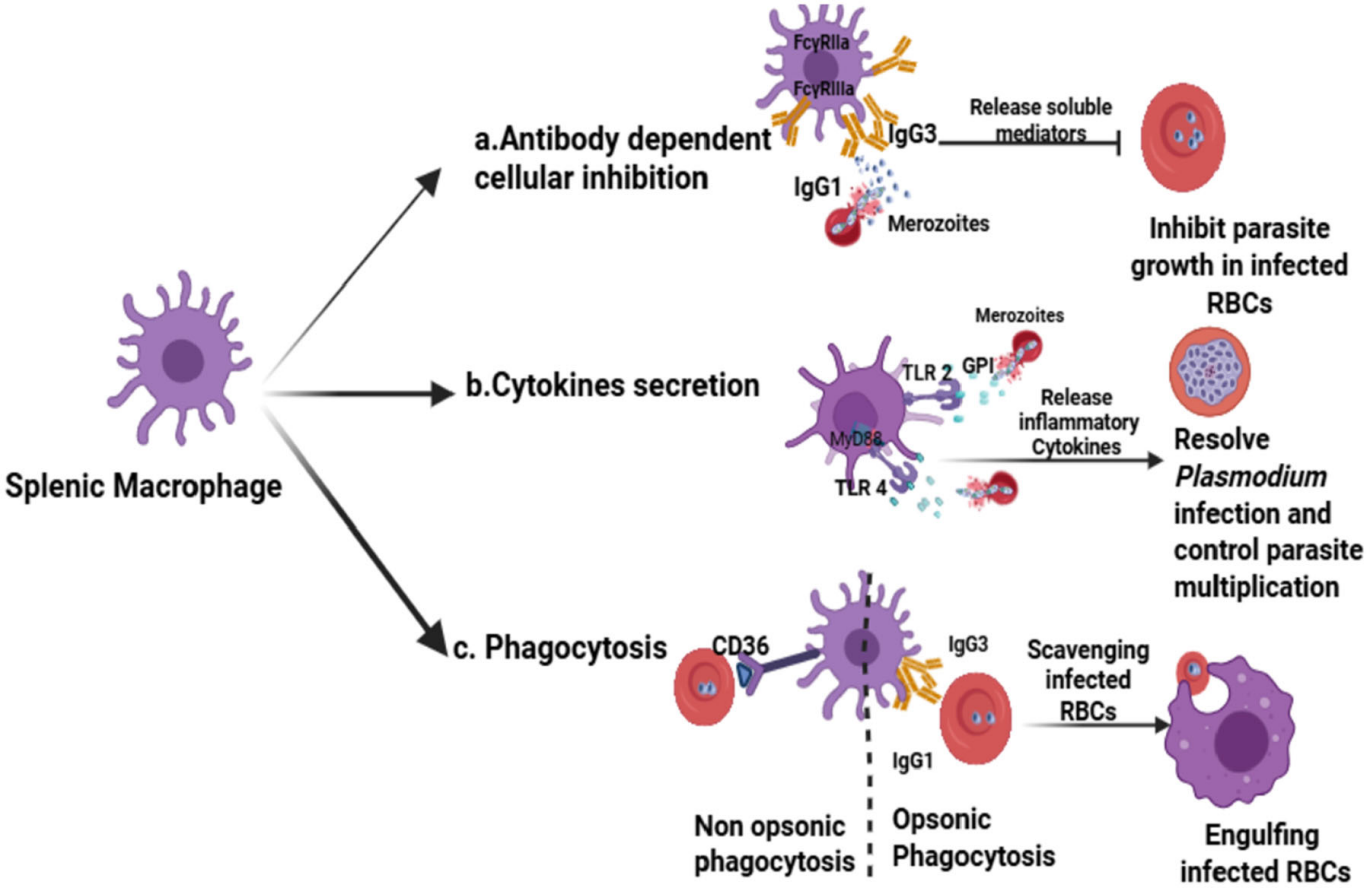
Immunological mechanisms by which splenic macrophages contribute to malaria pathogenesis control. (Created with Bio Render.com.)

Splenic macrophages play a pivotal role in immunity during malaria pathogenesis. They primarily contribute to immune defense and reduce the parasite control burden through their general action of mechanism, such as engaging in phagocytosis, facilitating antibody‐dependent cellular inhibition (ADCI), and producing a variety of cytokines [7, 36] as illustrate in Figure 2.

This schematic illustrates the multifaceted roles of splenic macrophages during *Plasmodium* infection, highlighting via three primary mechanisms: (1) *Antibody‐dependent cellular inhibition*: Splenic macrophages interact with opsonized merozoites via Fcγ receptors (FcγR), particularly FcγRIIa and FcγRIIIa, leading to the release of soluble mediators that inhibit parasite growth within iRBCs. (2) *Cytokine secretion*: Upon recognition of parasite‐derived molecules such as glycosylphosphatidylinositol (GPI) via Toll‐like receptors (TLR2 and TLR4), splenic macrophages secrete inflammatory cytokines that help control the parasite replication and promote resolution of infection. (3) *Phagocytosis*: Macrophages phagocytose iRBCs via both opsonic (IgG1/IgG3‐mediated) and non‐opsonic (CD36‐mediated) pathways, aiding in the scavenging and clearance of circulating infected parasitized RBCs.

Although splenic macrophage plays a significant function in the pathogenesis of malaria by engaging in several key processes, including initial activation of the innate immune response, changing in splenic microarchitecture and myeloid cell population, balancing of pro‐ and anti‐inflammatory cytokines through macrophage polarization, immunomodulation of immune response through erythropoiesis regulation and malarial anemia and phagocytosis of iRBCs. These key processes have been further discussed in detail below.

### 4.1 Splenic Macrophage Mediated Initial Activation of Innate Immune Response

Innate immunity serves as the initial barrier against pathogens, stimulating the production and release of various cytokines and chemokines that subsequently activate the innate immune response. Splenic macrophages are one of the important contributors of innate immune responses against malaria [36]. Innate immune sensing of malaria has been thoroughly reviewed by Gazzinelli et al. [80] and by Gowda et al. [81]. Splenic macrophages are equipped with a diverse array of pattern‐recognition receptors (PRRs) that are linked to pathogen‐associated molecular patterns (PAMPs) and damage‐associated molecular patterns (DAMPs) to effectively carry out their activities [82]. During *Plasmodium* infection, the interaction between pattern recognition receptors (PRRs) and PAMPs plays a crucial role in the activation of innate immune response against malaria [81]. Specifically, TLRs recognize various PAMPs present in *Plasmodium* parasites, triggering downstream signaling cascades that lead to the activation of pro‐inflammatory pathways. For instance, TLR9 is known to detect CpG motifs within plasmodial DNA [83], while TLR4 interacts with glycosylphosphatidylinositol (GPI) anchors on the surface of the parasite [84]. These interactions are pivotal in initiating the expression of inflammatory genes and proteins in macrophages, contributing to the overall generation of immune defense against the *Plasmodium* parasite [85, 86]. Thus, understanding the intricate interplay between PRRs and PAMPs in the context of *Plasmodium* infection sheds light on how the host's innate immune system recognizes and responds to the presence of the parasite, highlighting the importance of these molecular interactions in shaping the immune response during malaria.

In addition to PAMPs, Toll‐like receptors are also able to detect DAMPs at the site of tissue damage. For instance, endogenous damage signaling in stressed or necrotic cells causes secretion of proteins such as heat‐shock proteins (HSPs) that bind to TLR2 and TLR4 present on macrophages that leads to the production and activation of pro‐inflammatory cytokines and costimulatory molecules [87]. It has been demonstrated that infection with *Plasmodium* parasites causes the production of HSP homologs and that sepsis causes the release of HSPs into the bloodstream. Therefore, the heat‐shock response could serve as a significant in vivo signal during the differentiation process in the life cycle of the parasite [88, 89]. Moreover, TLRs may also recognize elements of the extracellular matrix like fibronectin and may result in macrophage activation in a similar manner to LPS stimulation [90]. Due to the substantial alterations in splenic microarchitecture after acute infection, the subsequent buildup of fibroblasts interior to the red pulp is generated during malaria [70]. Additionally, splenic macrophages also express NOD‐like receptor (NLR) family among the cytoplasmic PRRs [91]. For instance, disruption of the ionic gradient within the cell activates the member of the pyrin subfamily NLRP3, which leads to the formation of an inflammasome complex and, as a result, the production of the cytokines associated with inflammation, such as IL‐1 and IL‐18 [92]. Hemozoin has been shown to trigger NLRP‐3‐inflammasome in macrophages, a key event in the pathogenesis of rodent malaria [93, 94, 95] through Lyn and Syk kinases, resulting in the production of pro‐inflammatory cytokines [94]. Hemozoin also plays a significant role in inducing dysregulation of innate immunity, impairing host ubiquitination processes, and causing neuroinflammation and neurotoxicity. Due to phagocytosis of parasitized RBC, macrophages exhibit high levels of intracellular heme, which is crucial for neutralizing the toxic effects of hemoglobin [96]. Heme‐oxygenase 1 (HO‐1) is a crucial enzyme responsible for breaking down free heme, when present in excess, is toxic to macrophages [97]. In a rodent model, it was shown that when hemozoin is combined with parasite DNA, it was observed that the DNA–Hz complex prompted the translocation of TLR9, which served as both a priming and activation signal for inflammasomes. Following phagocytosis, Hz and DNA separate. Subsequently, Hz causes destabilization of the phagolysosome, permitting the contents to enter the cytosol, where DNA receptors are activated. Comparable findings were noted with RBCs infected by *P. falciparum*. Ultimately, the infected erythrocytes activated both the NLRP3 and AIM2 inflammasomes. These findings indicated that Hz and DNA collaborate to trigger systemic inflammation in the context of malaria, where it engages with the intracellular sensor TLR9. This interaction triggers an inflammatory response marked by the generation of pro‐inflammatory cytokines [93, 98]. All this concludes that macrophages are essential in the initial defense against malaria through recognition, phagocytosis, and activation of the broader immune response.

However, the timing of infection also plays a critical role in shaping the magnitude and effectiveness of these immune response. A recent study conducted by Cabral et al., in 2024 in which they revealed that circadian clock within immune cells, specifically macrophages, can modulate their reaction to infections, with variations in cytokine production and reactive oxygen species depending on the time of infection and affects the overall dynamics of malaria infections, impacting the host's susceptibility and the parasite's developmental cycle [99, 100]. Such evidence highlights the importance of both innate immune mechanisms and circadian biology during malaria pathogenesis.

### 4.2 Changes in Splenic Microarchitecture and Myeloid Cell Dynamics

The primarily intraerythrocytic nature of malarial parasites, which leads to significant alterations in the splenic microarchitecture during malaria infection [75, 101], makes the spleen a critical organ for studying host–parasite interactions [102, 103]. The hypothesis put forward by Weiss suggests that the blood–spleen barrier serves as a protective mechanism to safeguard developing erythroblasts and reticulocytes in the spleen's red pulp from the parasitic invasion. While shielding immature RBCs, this mechanism limits the ability of macrophages to access parasitized RBCs (pRBCs), thereby preventing them from clearing these infected cells at a critical phase of the infection. Furthermore, Weiss and other researchers [70, 104] observed that pRBCs tend to slow down and adhere in a rolling motion to the cells and results in the development or forming of the blood–spleen barrier. However, the specific proteins that mediating this cytoadherence remain unidentified.

In parallel, the critical changes in number of myeloid cell population occurring in the spleen play an important role during malaria infection. Additionally, the presence of macrophage migration inhibitory factor (MIF) in malaria infections has been linked to modulating monocyte recruitment and activation, impacting parasitemia levels in mice. During malaria infections, splenic macrophages play a crucial role in sustaining the production of granulocyte–macrophage colony‐stimulating factor (GM‐CSF) and interleukin‐3 (IL‐3) by specific subsets of IgM+ and IgG+ B1b B cell plasmablasts, showcasing their significance in the immune response to malaria [105]. These cytokines are produced initially by IgM+ B1b B cells and later by IgG+ plasmablasts, indicating a potential isotype switching during infection. The production of GM‐CSF and IL‐3 is partially dependent on Type I and Type II interferon signaling, emphasizing the complex interplay of various immune cells and mediators in response to *Plasmodium* infection. These sustained production of GM‐CSF and IL‐3 from B1b B cell plasmablasts underscores the phenotypic diversity and functional importance of these cells for mounting an effective immune response against malaria [106].

In addition, a recent murine malaria study using *P. chabaudi* infection, it was observed that the parasite triggered a significant expansion of macrophages and monocytes in the host. This myeloid expansion was shown to be dependent on both CD4⁺ T cells and the cytokine macrophage colony stimulating factor (MCSF). The upregulation of *Csf1* was found to be inducibly expressed in antigen‐experienced CD4⁺ T cells from infected mice, suggesting that MCSF produced by CD4⁺ T cells plays a critical role in parasitemia control and host recovery during the later stages of infection, to restrict the growth of the blood‐borne intracellular pathogen [107]. Although plasmacytoid DCs (pDCs), a population of leukocytes, are the most potent producer of Type I interferon contributes to the maintenance of immune homeostasis, and are essential for effective immune responses [108]. A study demonstrated that pDC upon priming by CD169^+^ macrophages, initiates Type I interferon (IFN‐I) secretion in the BM of malaria‐infected mice via cell‐intrinsic TLR7 sensing and cell‐extrinsic STING sensing which improves the outcomes of infections [49, 109, 110]. Furthermore, IL7‐R^+^c‐Kit^hi^ progenitors are primarily generated myeloid cells in infected host that are essential for the removal of infected erythrocytes. The development of these infection‐induced progenitors is largely dependent on the signaling of interferon‐γ (IFN‐γ) in hematopoietic progenitor cells. As a result, IFN‐γ plays a vital role in regulating hematopoiesis and both innate and adaptive immune responses during acute malaria infections [111]. Furthermore, Carvalho et al. in 2024 reported that alveolar macrophages contribute to malaria immunity by increasing macrophage sub‐population and their activation upon parasite exposure [112]. Together, these findings highlight the significance of myeloid cell dynamics and their regulation by cytokines and interferons in mediating effective host defense during malaria infection.

### 4.3 Balancing of Pro and Anti‐Inflammatory Cytokines by Splenic Macrophage

**Figure 3:**
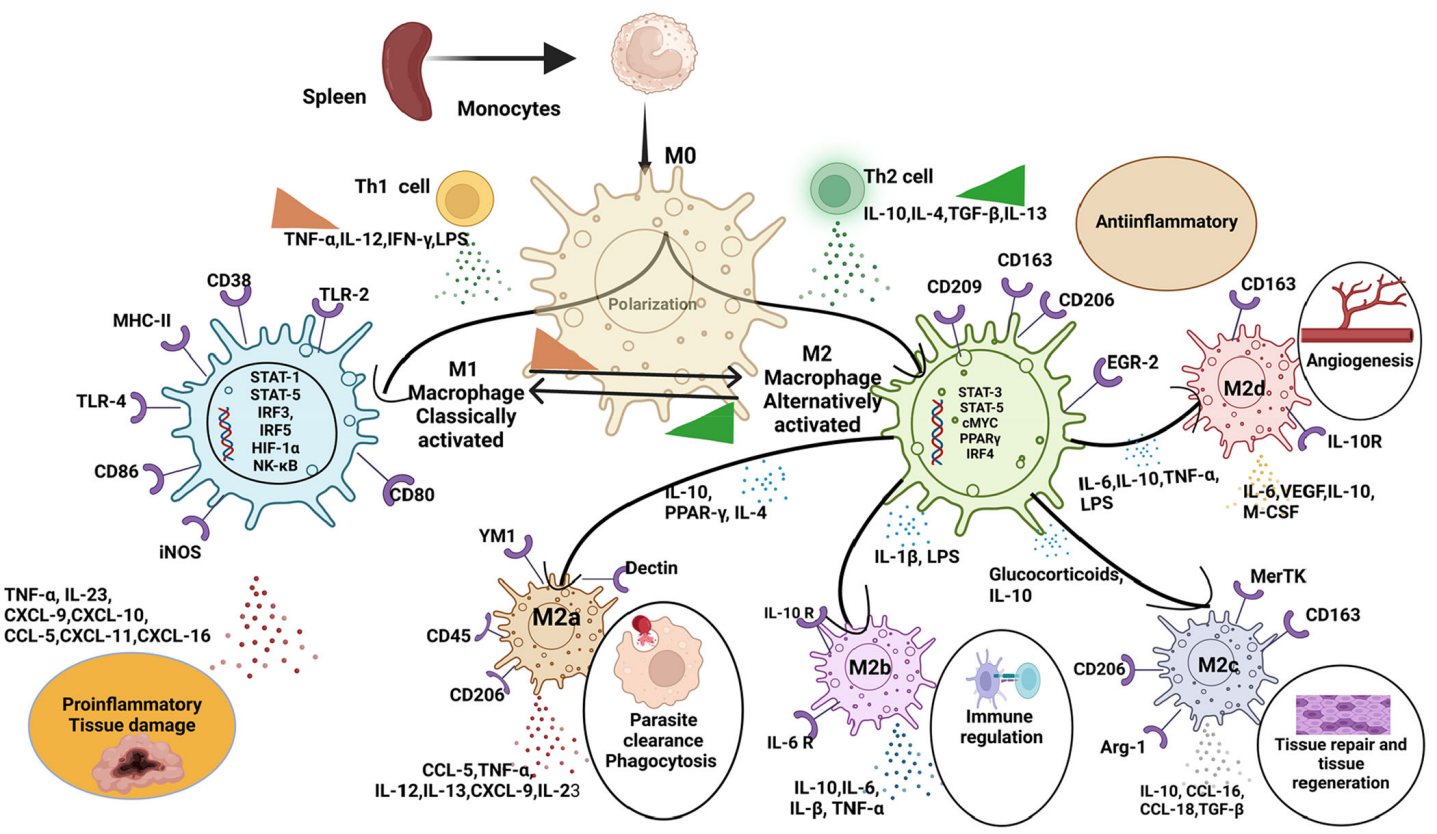
Continuum macrophage polarization spectrum of splenic macrophages during malaria pathogenesis, illustrating M1 and M2 subtypes characterized by distinct biomarkers and biological functions. Orange and green right triangles represent pro‐inflammatory and anti‐inflammatory cytokines levels, respectively. (Created with Bio Render.com.)

Macrophage polarization is dynamic, context‐dependent, and plays a crucial role in the host immune response, parasite control, tissue repair, and inflammation resolution [113, 114, 115, 116, 117]. The polarization of macrophage is influenced by various factors, including the types of cytokines produced, the duration of antigen exposure, and the availability of growth factors, fatty acids, prostaglandins, and molecules derived from pathogens. These factors initiate signaling pathways, notably the mTOR/PI3K/Akt pathway, which plays a crucial role in determining the macrophage activation phenotype. Additionally, alterations in microenvironmental stimuli can lead to a reversal of the initial polarization, highlighting the plasticity inherent in macrophages [118, 119]. In 2015, Mills and colleagues introduced a novel classification system that categorized macrophages in two groups: M1 and M2 [120] as shown in Figure 3. Depending on the activating stimuli in the microenvironment, classically activated macrophage (M1) are primarily derived from Th1 cytokines that are associated with pro‐inflammatory functions, including phagocytosis and microbicidal activity. On the other hand, alternatively activated macrophages (M2) activation is derived from Th2 cytokines that are involved in tissue remodeling and the resolution of inflammation [118, 121, 122]. Thus, the release of specific cytokines and chemokines from macrophages, along with the expression of various surface receptors, can drive alterations in the microenvironment that are critical for both innate and adaptive immune responses [123]. However, recent research has highlighted limitations in this understanding. The oversimplification of binary classification of the M1/M2 model fails to account for the spectrum of macrophage activation states, and the dynamic response to various stimuli, based on environmental cues and pathogen interactions, does not adequately capture [124]. Therefore, the evolving concept of the M1/M2 paradigm and has shifted toward a more nuanced, spectrum‐based continuum model that macrophage responses are highly plastic, influenced by complex combinations of cytokines, microbial signals, metabolic cues, and tissue‐specific factors. For instance, macrophages can simultaneously express markers of both M1 and M2 states, or switch phenotypes dynamically in response to changing microenvironmental conditions [125, 126, 127, 128, 129]. In the context of malaria, these evolving concepts are particularly intriguing, as they may provide insights into how different macrophage populations contribute to disease pathogenesis and influence the host immune response. Understanding these dynamics could lead to novel therapeutic strategies aimed at modulating macrophage activity, thereby enhancing the effectiveness of malaria treatments and vaccines. This approach underscores the importance of targeting specific macrophage subsets to achieve a balanced immune response, potentially improving outcomes in malaria and other infectious diseases.

This schematic illustration depicts the dynamic continuum polarization of splenic macrophages (M0) into classically activated (M1) and alternatively activated (M2) emphasizes the phenotypic and functional plasticity of splenic macrophages in mediating both host defense and immune regulation during malaria pathogenesis. Classically activated M1 macrophages, induced by Th1‐type cytokines (TNF‐α, IL‐12, IFN‐γ) and TLR ligands (e.g., LPS), express surface markers such as CD38, CD80, CD86, MHC‐II, TLR2, and TLR4. Their activation is governed by transcription factors including STAT1, STAT5, IRF3, IRF5, HIF‐1α, and NF‐κB, promoting the secretion of pro‐inflammatory cytokines (e.g., TNF‐α, IL‐23) and chemokines (e.g., CXCL9, CXCL10, CXCL11), contributing to parasite clearance but also causing collateral tissue damage. In contrast, alternatively activated M2 macrophages are driven by Th2‐associated cytokines (IL‐4, IL‐10, TGF‐β, IL‐13) and further diversify into subtypes with distinct immunological roles. M2a macrophages promote parasite clearance and phagocytosis; M2b macrophages regulate immune responses via secretion of IL‐10, IL‐1β, and TNF‐α; M2c macrophages are involved in anti‐inflammatory responses and tissue repair via secretion of IL‐10 and glucocorticoids; and M2d macrophages contribute to angiogenesis through VEGF, IL‐6, and M‐CSF production. These M2 subsets are regulated by key transcription factors such as STAT3, STAT6, PPARγ, cMYC, and IRF4.

Depending on the causative agent of infection, human parasites elicit unique polarization programs in macrophages that depend on the specific infectious agent, resulting in either M1 or M2 activation. Infections caused by protozoa, including *Leishmania*, *Toxoplasma*, *Trypanosoma*, and *Plasmodium*, generally initiate an M1 macrophage response. This initial response is vital for controlling parasitemia and managing the disease. Following this, there is frequently a shift from M1 to a partial M2 polarization, which helps reduce inflammation‐related tissue damage while allowing for the persistence of chronic infection [130]. Hence, a finely tuned balance between M1 and M2 is very crucial for an effective immune response. M1 polarization helps in the early control of parasite replication and activation of the immune response [131, 132, 133]. Pro‐inflammatory cytokines play a crucial role in managing intraerythrocytic *Plasmodium* infections in both humans and experimental animal models [134, 135]. During the initial phases of *Plasmodium* infection, activated innate immune cells release Type I Interferons which subsequently stimulate natural killer (NK) and natural killer T (NKT) cells to produce IFN‐γ [8]. An IFN‐γ dependent priming of monocytes is vital for enhancing resistance to infectious agents; however, it also leads to an excessive production of pro‐inflammatory cytokines in response to *Plasmodium*‐derived PAMPs and malaria‐associated DAMP. This turns to clinical features of malaria and its associated syndromes, including systemic inflammation, anemia, metabolic acidosis, and both cerebral and placental malaria [8].

As the infection progresses, macrophages can undergo alternative activation (M2 polarization) in response to anti‐inflammatory cytokines. M2 macrophages exhibit immune‐regulatory functions such as tissue repair through expression of scavenger receptors involved in tissue remodeling and resolution of inflammation by producing anti‐inflammatory cytokines (e.g., IL‐10, TGF‐β) during the later stages of malaria infection [131]. To mitigate the immunopathological effects of malaria, monocytes and macrophages increase the expression of M2‐associated immunoregulatory factors, such as Heme Oxygenase‐1 (HO‐1) [136] and IL‐10 [136] which reduce both oxidative burst and inflammation but simultaneously heighten the risk of bacterial superinfections, particularly with non‐typhoidal *Salmonella* [137]. Studies have shown that oxidative stress triggers inflammatory activation of macrophages, with xanthine oxidase‐produced reactive oxygen species inducing a strong cytokine response [138]. Additionally, antibody opsonization of *P. falciparum*‐infected erythrocytes activates the inflammasome in macrophages, leading to pro‐inflammatory cytokine secretion [139]. Furthermore, during acute malaria, monocytes display activated phenotypes and altered inflammatory transcriptional profiles, with impaired phagocytosis of infected erythrocytes observed [45]. In severe malaria, dysregulation of inflammatory mediators, such as IL‐12, IL‐10, TNF‐α, PGE2, and NO, occurs, impacting cytokine production and effector molecule balance [140]. A study in which administration of sgp130Fc recombinant chimera protein lowers the parasitemia, increases the survivability of *P. berghei* ANKA infected mice, and restores the distorted ratios of M1/M2 macrophage, mDC/pDC, and Th‐17/Treg by Trans IL‐6 signaling inhibition which reduce M1 macrophage during experimental cerebral malaria [141].

#### 4.3.1 Natural Compounds as Modulators of Macrophage Polarization and Inflammatory Responses During Malaria

Certain natural compounds have been found to influence the production of various inflammatory factors, either stimulating or inhibiting the inflammatory response. One such bioactive compound is curcumin, derived from the dried rhizomes of *Curcuma longa*, which has demonstrated pharmacological properties, including antitumor, antimicrobial, and antiangiogenic activities [142]. Additionally, curcumin derivatives exert significant cellular effects, such as regulating transcription factors like NF‐κB, modulating the expression of inflammatory cytokines, and influencing the production and activation of enzymes linked to infection progression [142], including the regulation of mTOR/PI3K/Akt involved in the macrophage activation phenotype [143, 144]. Experimental studies on mice infected with *Plasmodium* have shown that curcumin can effectively reduce parasitemia, enhance survival rates, and promote neuroprotection in cases of cerebral malaria [144]. This outcome is linked to the CD36‐mediated internalization by M2 macrophages, which facilitates the removal of neutrophils and dead tissue, aiding in inflammation resolution and parasite clearance. Similarly, another study revealed that mice treated with a peroxisome proliferator‐activated receptor‐γ (PPAR‐γ) agonist experienced reduced parasitemia and a dampened inflammatory response. This effect was also mediated by CD36, which acts as a ligand for *P. falciparum* erythrocyte membrane protein 1 (PfEMP‐1), expressed by the parasite [145]. Therefore, understanding the regulation of macrophage polarization and cytokine secretion is crucial for managing inflammation in malaria and improving therapeutic interventions.

### 4.4 Splenic Macrophage in Erythropoiesis Regulation and Malarial Anemia

It is well known that erythrocyte production and destruction both are tightly controlled by macrophages [146]. During steady‐state haematopoiesis, human erythroblastic islands produce approximately 10^10^ erythrocytes per hour. Among these erythroid islands, local myeloid macrophages provide erythroblasts interactions with cells which are critical to erythrocyte formation. Moreover, recent investigation indicates that macrophages also significantly contribute in the process of erythropoiesis by promoting the differentiation and maturation of nucleated RBCs into reticulocytes, as well as removing old RBCs from circulation [147]. All these highlighted the significance of molecular connections between erythroid progenitor cells and central macrophages in maintaining the function and integrity of erythroblastic island [148].

According to Bessis and Breton‐Gorius (1962), macrophages may stimulate erythropoiesis through the delivery of iron directly to erythrocyte progenitor cells, therefore directly contributing to the process of erythropoiesis [149]. Splenic RPMs and liver KCs are the two main contributors to this iron supply in the body. The primary source of iron within a macrophage is acquired through the process of erythrophagocytosis, where macrophages identify and break down senescent RBCs within their lysosomes [149]. Additionally, *Spi‐c*, a transcription factor plays a role in preserving the iron processing capabilities of RPMs in the spleen and nurse macrophages in the BM [28]. Central nurse macrophages located in the BM contribute to erythropoiesis within the erythroblastic island niche, where they primarily utilize iron bound to transferrin. Therefore, these nurse macrophages are crucial for supporting the development of erythroblasts by regulating their proliferation and differentiation, controlling the subsequent release of reticulocytes into the bloodstream [150]. Although splenic RPMs are primarily responsible for the re‐emergence of iron that results from the recycling of old and destroyed RBCs in the spleen. Ferritin, an iron reservoir protein generated by macrophages through the process of exocytosis, while macrophages are responsible for sequestration of hemoglobin in BM [151]. This ferritin is taken up by erythroblasts through endocytosis in islet culture of erythroblasts where iron is liberated from ferritin by acidification and proteolysis which subsequently becomes accessible for the synthesis of heme inside the erythroid precursor cell [151, 152, 153, 154]. Additionally, splenic macrophages are part of the erythroblastic islands within the spleen, where they interact with developing erythroid cells to promote erythropoiesis. Erythroblastic island macrophages express the erythropoietin receptor (Epor) and play a role in supporting erythropoiesis by responding to erythropoietin signaling, which is essential for the differentiation of stress erythroid progenitors [155]. Despite our understanding of the interactions between macrophages and erythroblasts in erythroblast islets and their function in erythroblast formation is growing, most of the numerous research investigations had been performed in vitro. In a study, Chow and colleagues deftly demonstrated that CD169 macrophages stimulate erythropoiesis in vivo, both under normal circumstances as well as during exposure to stress [156]. CD169 was first identified as a marker of central macrophages in erythroblastic islets. CD169–CD43 interaction is involved in erythroblastic island formation and erythroid differentiation. Reduced erythroblast cells in the BM and moderate iron deficiency anemia are consequences of CD169 macrophage depletion [156, 157].

Linking these roles to malaria, malaria anemia is a severe complication arising from malaria infection that occurs when the parasite destroys the healthy RBCs [158, 159]. Studies have shown that Toll‐like receptor 7 (TLR‐7) promotes splenic erythropoiesis in *P. yoelii* NSM‐infected mice through the regulation of iron metabolism of macrophages by enhancing the production of IFN‐γ, which in turn boosts the phagocytosis of infected erythrocytes by macrophages [160]. This process highlights the dual role of splenic macrophages in both clearing the parasite and supporting erythropoiesis, and ensures that the spleen can respond effectively to both the infectious challenge and the resulting anemia. MIF is a pleiotropic cytokine involved in immunomodulatory functions of several inflammatory disorders, such as sepsis [161], inflammatory bowel disease [162], allergic [163], rheumatoid arthritis, and lupus [164]. Moreover, MIF is also involved in the protection of several parasitic infections [165, 166], such as *Taenia crassiceps* [167] and *Toxoplasma gondii* [168]. MIF has a significant role in controlling the immune response linked to host pathogenesis and lethality, which is vital to consider while attempting to prevent or mitigate the consequences of *Plasmodium* infection. Some research suggests that *Plasmodium* infection increases MIF levels in humans and mice, indicating that the pro‐inflammatory response triggered by MIF may lead to pathology, severe malaria, and fatal outcomes [169, 170]. Another research study showed that low MIF production enhances the infection's severity during *Plasmodium* infection [171, 172, 173]. Furthermore, macrophages release MIF in response to malarial pigment, which inhibits erythroid progenitor‐derived colony formation, potentially impacting erythropoiesis and contributing to the pathophysiology of malaria anemia [174, 175].

### 4.5 Erythrophagocytosis of Infected RBCs by Splenic Macrophages

Macrophages also contribute to innate immunity through phagocytosis in malaria. In 1908, Elie Metchnikoff and Paul Ehrlich discovered macrophages and their phagocytosis function for the first time [176]. During *Plasmodium* infection, macrophages are responsible for recognizing and phagocytosing these parasitized iRBCs, leading to their destruction. This process results in clearing the parasite from circulation and preventing the parasitized RBCS to further invasion in other organs [3].

Splenic macrophages are responsible for the elimination of infected parasitized erythrocytes through the process of phagocytosis, contributing to the control of blood‐stage infection *P. yoelii* infection in iron‐deficit mice [177]. Additionally, splenic macrophages play an essential role in removing unwanted lesions from the erythrocyte membrane [178]. According to the ultrastructural examinations of primate's spleen infected with *Plasmodium knowlesi*, the phagocytosis ability of the spleen plays a crucial role in eradicating malaria parasites from iRBCs [3]. Additionally, noteworthy research by Buffett and colleagues utilizing perforated human spleens as testimony has demonstrated that elimination and clearance of malaria parasites from erythrocytes takes place in the red mass of the spleen [34]. According to several studies, the spleen plays a significant defensive role in the treatment of naïve patients because of its protective function. They clearly demonstrated that splenectomy enhanced severity of the disease, parasitemia, and fatalities [179, 180]. This whole process of erythrophagocytosis is mediated by the expression and interaction of two important surface molecules namely CD47 and SIRP‐α. CD47 (cluster of differentiation 47) protein is widely expressed on many cell types, including RBCs. SIRP‐α (macrophage‐expressed immunoreceptor signaling regulatory protein alpha) is well‐known for being capable to prevent phagocytosis of CD47‐expressing cells [181, 182]. In erythrocytes, the CD47‐ SIRP‐α interaction generates an intense negative signal during phagocytosis, therefore, can serve as a “self” marker. A “self” particle, such as an erythrocyte, is not phagocytosed by splenic macrophages because it has CD47 and the associated inhibitory signals brought on by exposure to macrophage SIRP‐α. Indeed, a minimal level of opsonization might be adequate to initiate phagocytosis of a foreign particle that lacks CD47 expression. Interestingly, patients with Rh‐null or protein 4.2 deficiency, both of which have markedly decreased CD47 expression levels on erythrocytes, have been reported to have mild hemolytic anemia, indicating toward the important role of CD47 molecule [183, 184]. Another study revealed that erythrocyte preservation led to the decrease of CD47, which may be correlated with more quickly blood cell clearance following transfusion [185]. On the other hand, a recent study revealed that CD47 may also serve as a “eat me” signal in addition to its role as a “don't eat me” signal [186]. It has also been demonstrated that the CD47‐SIRP‐α interaction allows a subset of aged erythrocytes found in the entire bloodstream to bind and be phagocytosed. In addition, there is evidence that experimental erythrocyte senescence produces conformational changes in CD47, turning it from an antagonistic to an activating molecule. Overall, the interaction between CD47 on RBCs and SIRPα on macrophages is crucial in regulating erythrophagocytosis in malaria [187]. Alterations in CD47 expression on infected RBCs result in increased phagocytosis by macrophages and contribute to disease complications such as anemia and splenomegaly [188]. Therefore, understanding and targeting CD47‐SIRP‐α interaction could offer new therapeutic strategies for managing malaria and its associated symptoms.

## 5 Conclusion

Splenic macrophages are increasingly recognized as crucial players in both malaria pathogenesis and immunity. Their role in controlling parasite load, modulating immune responses, and maintaining splenic architecture underlines their dual function in host defense and disease progression. Recent studies highlight that different macrophage subsets and their ability to phagocytose iRBCs and regulate inflammatory responses, positioning them as key modulators in the balance between effective immunity and immunopathology. Splenic macrophages play a critical role in malaria pathogenesis, with significant functions impacting disease progression, treatment strategies, and patient outcomes. The efficiency of splenic macrophages in clearing *Plasmodium*‐infected erythrocytes is directly linked to malaria severity, as effective macrophage responses correlate with lower parasitemia and milder disease, while compromised function can lead to severe complications like cerebral malaria and anemia. Additionally, the cytokine production by these macrophages influences the inflammatory environment, where excessive inflammation driven by cytokines can result in severe complications. *Plasmodium* parasites have evolved their mechanisms to evade detection by splenic macrophages, such as altering surface proteins and manipulating host immune responses. That is why there is a need of further research in this area which is crucial for developing treatments to prevent chronic infection. Therapeutically, enhancing macrophage functions through drugs or modulating cytokine production can improve parasite clearance and patient outcomes, while anti‐inflammatory therapies can help manage severe inflammation without compromising the immune response. In the context of vaccination and immunotherapy, understanding the antigen‐presenting role of splenic macrophages can help in better strategies for vaccine development to induce stronger immune responses, and immunotherapies that harness these macrophages' functions offer potential support to conventional treatments.

## 6 Future Clinical and Research Aspects

The information available in this review article comprises several original research studies which highlight the crucial role and importance of splenic macrophages in malaria pathogenesis and immunity. However, the immune potential of splenic macrophages during *Plasmodium* infection has not been fully characterized. While animal models have significantly advanced our understanding of splenic macrophages in malaria, translating these findings to human contexts remains a crucial step. However, differences in macrophage phenotypes and functions between humans and animals underscore the need for translational research and the development of innovative models that accurately reflect human biology. Current research on human splenic macrophages reveals several knowledge gaps, primarily due to the complexity of macrophage subpopulations, their interactions with other immune cells, and the influence of environmental factors on their behavior. These challenges hinder a comprehensive understanding of the role of human splenic macrophages in immune response. By bridging this gap, we can better understand the role of splenic macrophages in malaria pathogenesis and develop more effective therapeutic strategies. Therefore, new investigations are essential to elucidate the mechanisms by which splenic macrophages combat *Plasmodium* infection. This understanding could pave the way for the development of cell‐based immunotherapies.

Numerous studies indicate that macrophage‐based immunotherapies can be optimized by targeting specific macrophage functions. Macrophages are well known for their exhibiting remarkable plasticity, adapting their functions in response to various stimuli. This adaptability allows them to perform diverse roles, from pathogen clearance to the regulation of adaptive immunity. Recent investigations into various human diseases, including inflammatory arthritis, diabetic heart disease, chronic obstructive pulmonary disease (COPD), and cancer, have highlighted the potential of macrophage‐targeted immunotherapy to improve immune protection and disease outcomes by focusing on particular macrophage functions, and thereby enhancing vaccine formulations to promote strong and enduring immunity. In inflammatory arthritis, the therapeutic strategies aimed at repolarizing pro‐inflammatory macrophages M1 to the anti‐inflammatory phenotype M2 have been proposed as an effective approach to address the M1/M2 imbalance, thereby alleviating chronic inflammation and facilitating tissue repair [189]. These such mechanisms governing macrophage plasticity are fundamental for the success of novel macrophage targeting therapeutics. Furthermore, in the case of recurrent chronic lung disease‐ chronic obstructive pulmonary disease (COPD) and diabetic heart disease, emphasize the importance and role of macrophages as an innate immune cell to exacerbate inflammation by inducing inflammatory responses through activating both innate and adaptive immune responses, thereby accelerating disease progression [190, 191, 192]. In cancer, the impact of the tumor microenvironment on macrophage behavior is crucial for the advancement of effective therapies aimed at reprogramming macrophages toward an antitumor phenotype [193]. Thus, understanding the heterogeneity of macrophage subtypes complicates therapeutic targeting, necessitating further research to identify specific markers and pathways for intervention [193, 194, 195]. These research findings provide novel insights into the pivotal role of macrophages and emphasize the potential of macrophage‐targeted therapies. By modulating macrophage activity, these therapies offer promise for improving patient outcomes, advancing our understanding of disease mechanisms, and serving as a foundation for the development of effective vaccine strategies. However, the diversity among macrophage subtypes poses challenges for therapeutic targeting, underscoring the need for further research to identify specific markers and pathways for intervention. These discoveries pave the way for new avenues in research and clinical innovation, solidifying macrophages as critical targets in the pathogenesis, pathology, diagnosis, and treatment of various diseases.

In malaria, targeting splenic macrophages can be a promising strategy for developing new malaria therapies. Research shows that splenic RPMs can produce significant amounts of Type I interferons (T1‐IFNs) in response to malaria infection, highlighting their role in the immune response [196, 197]. Moreover, understanding the interactions of macrophages with malaria antigens can be essential for vaccine development. In addition, the targeted delivery of antigens to antigen‐presenting cells (APCs) can be represented as a promising strategy in the advancement of development of vaccination strategies. In this regard, CD169^+^ macrophages in the spleen have been recognized as crucial for inducing strong humoral immune responses, making them an attractive target for antigen delivery in vaccine strategies. CD169^+^ macrophages located in the marginal zone of the spleen are ideally positioned for antigen (Ag) delivery due to their strategic placement, which allows them to efficiently capture blood‐borne antigens. Their proximity to B cells and T cells in the white pulp promotes strong, isotype‐switched, high‐affinity antibody production, as well as the preferential induction and long‐term persistence of Ag‐specific germinal center (GC) B cells and follicular T helper (Th) cells. Supporting these observations, CD169^+^ macrophages have been shown to retain intact Ag, facilitate the activation of cognate B cells, and enhance the expression of costimulatory molecules upon activation, underscoring the potential of targeting CD169 for improved vaccine responses [198]. Furthermore, it is essential to emphasize on the induction of regulatory macrophages, which likely alters the immune microenvironment, dampening the robust innate and adaptive immune responses might be needed for effective immunity. Research on the interactions between human antigen‐presenting cells and sporozoites, as well as circumsporozoite proteins, has revealed that *Plasmodium* sporozoites possess the capability to inhibit immune responses through the induction of regulatory macrophages. The insights gained from these original research findings offer novel insights into the mechanisms of early immune regulation utilized by *Plasmodium* parasites, potentially elucidating the reasons behind the diminished protective efficacy observed with intradermal vaccination employing whole attenuated sporozoites [199]. Thus, exploring the intricate interactions of splenic macrophages with malaria pathogens will not only enhance our knowledge of immune responses but also provide critical information for innovative therapeutic and preventive measures against malaria. Therefore, a comprehensive understanding of targeting splenic macrophages in vaccine design could significantly enhance immune responses against malaria, making these macrophages a promising target for malaria vaccine development.

## Author Contributions

A.G. and J.D. designed the original content of the manuscript and developed it in detail. A.G. reviewed the literature, wrote the manuscript, generated the figures, and arranged references. M.K. provided valuable feedback that helped to revise the manuscript. All authors contributed to the article and approved the submitted version.

## Ethics Statement

The authors have nothing to report.

## Consent

The authors have nothing to report.

## Conflicts of Interest

The authors declare no conflicts of interest.

## Supporting information


**Figure:** Flow chart of article selection process with search strategy and keywords terms.

## Data Availability

The authors have nothing to report.
