# Supplementary material for: Emerging Role of Splenic Macrophage in Malaria Pathogenesis and Immunity
Source: Immun Inflamm Dis. 2025 Oct 31;13(10):e70258. doi: 10.1002/iid3.70258 (PMC12577441; doi:10.1002/iid3.70258)
Supplement: Supplementary file 1 — Figure: Flow chart of article selection process with search strategy and keywords terms. [file IID3-13-e70258-s001.docx]

PubMed Central and Google Scholar

(1955-2025)

| Search Keywords | PubMed Central | Google Scholar | Total |
| --- | --- | --- | --- |
| Developmental origins and differentiation of Splenic Macrophages | 4736 | 20,100 | \| 24836 \| \| --- \| |
| Splenic macrophage subtypes in malaria. | 2033 | 11,400 | 13,433 |
| Splenic macrophage phenotypes in malaria | 6734 | 16,800 | 23,534 |
| *Plasmodium* strain impact on splenic macrophages | 2550 | 17,000 | 19,550 |
| Splenic macrophage in erythropoiesis and malarial anaemia | 464 | 7,450 | 7,914 |
| Innate immune activation by splenic macrophages during malaria | 6601 | 16,200 | 22,801 |
| Splenic microarchitecture and myeloid cell dynamics in malaria | 33 | 172 | 205 |
| Circadian regulation of macrophage responses in malaria | 812 | 5,890 | 6,702 |
| M1/M2 Macrophage polarization in malaria | 438 | 1,210 | 1,648 |
| Natural compounds modulating macrophage polarization during malaria | 2483 | 16,300 | \| 18,783 \| \| --- \| |
| Erythrophagocytosis by Splenic Macrophages during malaria | 512 | 2,100 | 2,612 |
| CD47 -SIRP-alpha in malaria | 68 | 2,550 | 2,618 |
| Macrophage-based immunotherapies in disease including malaria | 32 | 102 | 134 |

The selection process involved purposive screening of articles according to predefined inclusion and exclusion criteria.

Inclusion criteria: articles on with suggested Keyword search terms indicated in above table.

Exclusion criteria: articles other than as described in inclusion criteria.

Additional Figure: Flow chart of article selection process with search strategy and keywords terms.
